# Genome Sequences and Methylation Patterns of Natrinema versiforme BOL5-4 and Natrinema pallidum BOL6-1, Two Extremely Halophilic Archaea from a Bolivian Salt Mine

**DOI:** 10.1128/MRA.00810-19

**Published:** 2019-08-15

**Authors:** Priya DasSarma, Brian P. Anton, Satyajit L. DasSarma, Fabiana L. Martinez, Daniel Guzman, Richard J. Roberts, Shiladitya DasSarma

**Affiliations:** a Institute of Marine and Environmental Technology, Department of Microbiology and Immunology, University of Maryland, Baltimore, Maryland, USA; b New England BioLabs, Ipswich, Massachusetts, USA; c Instituto de Investigaciones para la Industria Química, Consejo Nacional de Investigaciones Científicas y Técnicas, Universidad Nacional de Salta, Salta, Argentina; d Centro de Biotecnología, Faculty of Sciences and Technology, Universidad Mayor de San Simón, Cochabamba, Bolivia; Georgia Institute of Technology

## Abstract

Two extremely halophilic archaea, namely, Natrinema versiforme BOL5-4 and Natrinema pallidum BOL6-1, were isolated from a Bolivian salt mine and their genomes sequenced using single-molecule real-time sequencing. The GC-rich genomes of BOL5-4 and BOL6-1 were 4.6 and 3.8 Mbp, respectively, with large chromosomes and multiple megaplasmids. Genome annotation was incorporated into HaloWeb and methylation patterns incorporated into REBASE.

## ANNOUNCEMENT

Halophilic microbes capable of surviving extreme conditions are of interest for biotechnology and astrobiology (1, 2). Two extremely halophilic archaea, members of the *Natrinema* genus, were isolated from pink salt obtained from a salt mine in the Department of Tarija, O’Connor Province, Bolivia. Together with the novel *Halorubrum* sp. strain BOL3-1 from Salar de Uyuni, our collection of halophilic archaea from Bolivia has expanded from one to three species (3). These strains also provide the basis for a wider comparative genomic analysis of haloarchaea from the subsurface to high elevation.

Pink salt was sampled at a remote salt mine (21°24′19.73″S, 64°07′51.52″W) at 1,230-meter elevation, where temperatures range from −10°C to 37°C. The salt samples were dissolved in CM^+^ medium, and growth was stimulated with shaking at 220 rpm at 37°C under illumination (4). The enrichment cultures were plated on CM^+^ agar plates and two isolates, namely, Natrinema versiforme BOL5-4, a nearly unpigmented strain, and Natrinema pallidum BOL6-1, a pigmented strain, were purified by 3 rounds of streaking.

Nucleic acids were extracted using a standard method (5), and sequencing was performed using the Sequel platform (Pacific Biosciences, Menlo Park, CA). SMRTbell libraries were prepared from unsheared genomic DNA (2 μg BOL5-4 and 0.9 μg BOL6-1), and each library was sequenced on 1 single-molecule real-time (SMRT) cell with a Sequel binding kit 3.0, with 10-h collection and 2-h preextension times (6). Sequencing subreads were filtered and assembled *de novo* using Hierarchical Genome Assembly Process (HGAP) version 4, with default parameters. There were 553,417 filtered subreads (mean length, 4,931 bp; coverage, 780×) for BOL5-4 and 551,878 filtered subreads (mean length, 4,717 bp; coverage, 520×) for BOL6-1.

The *N. versiforme* BOL5-4 and *N. pallidum* BOL6-1 genome sequences were annotated using the NCBI Prokaryotic Genome Annotation Pipeline (PGAP) build 3190 (7), which was incorporated into HaloWeb (version r1559404112; https://halo.umbc.edu/) and analyzed further using EMBOSS version 6.6.0.0 (http://www.bioinformatics.nl/cgi-bin/emboss/) (8). Default parameters were used for all software. The BOL5-4 genome was 4,674,473 bp (G+C content, 63.4%) and included a 3,747,116-bp circular chromosome (G+C content, 64.7%) and 4 plasmids, namely, pNVE500 (492,102-bp linear contig; G+C content, 56.3%), pNVE414 (413,865-bp circle; G+C content, 60.0%), pNVE19 (18,925-bp circle; G+C content, 63.5%), and pNVE2 (2,465-bp circle; G+C content, 69.2%). The BOL6-1 genome was 3,778,093 bp (G+C content, 64.3%) and included a 3,503,953-bp circular chromosome (G+C content, 64.6%) and 2 plasmids, namely, pNPA200 (203,201-bp circle) and pNPA70 (70,939-bp circle), both with a G+C content of 60.6%.

The BOL5-4 genome contained 4,589 genes, including 3 rRNA operons and 64 tRNA genes, whereas the BOL6-1 genome contained 3,785 genes, including 3 rRNA operons and 50 tRNA genes. Both proteomes were highly acidic (9), with calculated mean pI values of 4.6 to 4.7, and all but 5 of nearly 800 core haloarchaeal orthologous groups were encoded in the genomes (10, 11). Both contained expanded gene families, e.g., Orc/Cdc6, TATA-binding, and transcription factor B (TFB) genes (12), as well as a gene cluster for gas vesicle nanoparticles (13) and polyhydroxyalkanoate synthesis genes (14). Both genomes encode many transposases, namely, a total of 100 in BOL5-4 and 80 in BOL6-1 (15).

Methylated DNA motifs and the methyltransferases (MTases) predicted to be responsible for some were deposited in REBASE (Table 1) (16). Both genomes contained the methylated motifs ^m4^CTAG and C^6m^ATTC, which are common to halophilic archaea.

**TABLE 1:** Motifs containing methylated bases ^m6^A and ^m4^C

Motif^*a*^	*N. versiforme* BOL5-4		*N. pallidum* BOL6-1	
	% modified	Gene^*b*^	% modified	Gene^*b*^
GACGA**A**C	100	FEJ81_15005		
C**A**TTC	100	FEJ81_07280	99.9	FGF80_04050
C**C**WGG	99.3	FEJ81_16560		
GAAC**A**YC	100	FEJ81_15230		
**C**TAG	97.0	FEJ81_09745	98.7	FGF80_01935
TCCT**C**GG	96.0	FEJ81_19855		
GCA**A**T	71.4	FEJ81_20490		
GTAYT**C**G			98.8	FGF80_00870
CAGYA**A**C			100	FGF80_10950

a Locations of methylated bases are in bold for the top strand and underlined for the bottom strand.

b Putative assignments of MTases responsible for the modification in the first column based on sequence comparison with known enzymes of that specificity in REBASE.

### Data availability.

The *N. versiforme* BOL5-4 genome sequence has been deposited in GenBank with the accession numbers CP040329, CP040330, CP040331, CP040332, and CP040333. Raw data are available in the NCBI Sequence Read Archive with the accession number SRX5888851. The *N. pallidum* BOL6-1 genome sequence has been deposited in GenBank with the accession numbers CP040637, CP040638, and CP040639. Raw data are available in the NCBI Sequence Read Archive with the accession number SRX6057204.
